# The Burden of Antimicrobial‐Resistant *Pseudomonas aeruginosa* Isolates in Children With Cystic Fibrosis: Molecular Characterization and Genotyping Analysis

**DOI:** 10.1002/mbo3.70217

**Published:** 2026-01-28

**Authors:** Erfaneh Jafari, Babak Pourakbari, Mohammad Reza Asadi Karam, Reza Azizian, Mohammad Reza Modaresi, Setareh Mamishi

**Affiliations:** ^1^ Pediatric Infectious Diseases Research Center (PIDRC) Tehran University of Medical Sciences (TUMS) Tehran Iran; ^2^ Department of Molecular Biology Pasteur Institute of Iran Tehran Iran; ^3^ Department of Pediatrics School of Medicine Tehran University of Medical Sciences (TUMS) Tehran Iran

**Keywords:** carbapenem resistance, children, cystic fibrosis, genotyping, multidrug resistance, *Pseudomonas aeruginosa*

## Abstract

*Pseudomonas aeruginosa* poses a significant therapeutic challenge in pediatric patients with cystic fibrosis (CF) due to increasing multidrug resistance (MDR) and carbapenem resistance, underscoring the need for surveillance to guide treatment strategies. In this study, sputum and throat swab samples were collected from inpatient and outpatient CF children with pulmonary infection at the Children's Medical Center in Tehran, Iran. Isolates were identified using standard culture and biochemical methods, followed by antimicrobial susceptibility testing. Carbapenemase production was assessed phenotypically and by molecular detection of resistance genes, and genetic diversity was also evaluated using Random Amplified Polymorphic DNA (RAPD)–polymerase chain reaction (PCR). A total of 117 *P. aeruginosa* isolates were recovered (prevalence 17.41%), of which 94.9% were nonsusceptible to at least one antimicrobial agent. Carbapenem‐resistant *P. aeruginosa* (CRPA) and MDR isolates accounted for 24.8% and 23.1% of isolates, respectively. Carbapenemase gene coexistence was significantly associated with MDR (*ρ* = 0.227, *p* = 0.014) and CRPA (*ρ* = 0.314, *p* = 0.001). Metallo‐β‐lactamase production was detected in 13.7% of isolates, while *blaVIM* was the most frequently identified carbapenemase gene (59%). RAPD–PCR demonstrated marked genetic heterogeneity, grouping isolates into 24 distinct clusters. Overall, the substantial burden of MDR and CRPA identified at this tertiary pediatric center highlights an urgent need for stricter antimicrobial stewardship, enhanced infection control measures, and ongoing surveillance to mitigate resistance spread and preserve therapeutic effectiveness in this vulnerable population. These findings warrant multicenter investigation to determine whether similar patterns exist across other Iranian pediatric CF facilities.

## Graphical abstract

This study highlights the prevalence of multidrug‐ and carbapenem‐resistant *Pseudomonas aeruginosa* in pediatric cystic fibrosis patients, along with the presence of carbapenemase‐encoding genes and genetic profiles, suggesting clonal spread. These findings underscore the urgent need for surveillance and strategies to combat resistant strains and improve treatment outcomes.

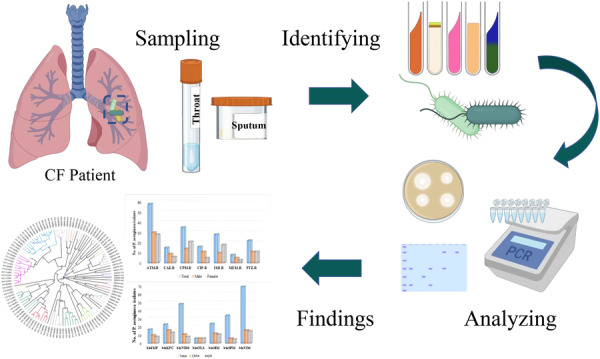

**Abbreviations**
AMR: antimicrobial resistance
AORs: adjusted odds ratios
CDDST: combined double‐disk synergy test
CF: cystic fibrosis
CFUs: colony forming units
CIs: confidence intervals
CLSI: Clinical and Laboratory Standards Institute
CR: carbapenem resistant
CRPA: carbapenem‐resistant *P. aeruginosa*
MBL: metallo‐β‐lactamase
MDR: multidrug resistant
MLST: multilocus sequence typing
OPD: outpatient department
PCR: polymerase chain reaction
*P. aeruginosa*
: *Pseudomonas aeruginosa*
RAPD–PCR: random amplified polymorphic DNA‐PCR
*S. aureus*
: *Staphylococcus aureus*
WGS: whole‐genome sequencing
WHO: World Health Organization

## 1 Introduction

Cystic fibrosis (CF) is a genetic disorder causing thick, sticky mucus in children's lungs, airways, and digestive system. This mucus blocks airways, trapping bacteria, leading to frequent lung infections and progressive lung damage. Early symptoms include coughing, breathing difficulties, and poor nutrient absorption, causing malnutrition. Notably, *Pseudomonas aeruginosa* (*P. aeruginosa*) infection is common in CF children, leading to chronic lung colonization, persistent inflammation, accelerated lung function decline, and increased morbidity and mortality (Burgel et al. 2024; Nickerson et al. 2024).

In the United States, approximately 25% of individuals with CF were infected with *P. aeruginosa* by 2023. The management of CF is particularly challenging in the presence of this opportunistic pathogen, owing to its remarkable adaptability and propensity to develop resistance to multiple classes of antibiotics. In 2024, the World Health Organization (WHO) revised its list of priority bacterial pathogens, emphasizing species with significant antimicrobial resistance (AMR). *P. aeruginosa* was designated as a high‐priority pathogen due to its impact in healthcare settings (WHO 2024; CF‐Foundation 2023; CDC 2024).

The limited permeability of the outer membrane gives *P. aeruginosa* inherent resistance to multiple classes of antibiotics. The pathogen can further augment its resistance profile via mutational changes or acquisition of resistance genes, leaving few effective treatment options. Particularly, the production of class B β‐lactamases is a common mechanism underlying carbapenem resistance (CR) in *P. aeruginosa* (Glen and Lamont 2021; Langendonk et al. 2021; Elfadadny et al. 2024).

Understanding the genetic variation and relatedness of bacterial strains involved in CF infections is crucial for tracing transmission routes and implementing effective infection‐control measures. A variety of molecular typing techniques are widely used for this purpose. Random Amplified Polymorphic DNA (RAPD)–polymerase chain reaction (PCR) represents one such method, offering a practical and economical approach for differentiating bacterial strains in resource‐limited settings (Abdel‐Rhman and Rizk 2021; Bogiel et al. 2022). However, in pediatric CF populations from Iran and the broader Middle East, studies integrating phenotypic antimicrobial susceptibility profiles with molecular detection of carbapenemase‐associated genes and strain‐typing data remain scarce. This gap limits accurate interpretation of local AMR epidemiology and potential transmission dynamics (Mamishi et al. 2023; Rastegar‐Kashkouli et al. 2024).

The objective of this research was to analyze resistance patterns and genetic variability among *P. aeruginosa* isolates from pediatric CF patients, thereby enhancing understanding of multidrug‐resistant (MDR) and carbapenem‐resistant *P. aeruginosa* (CRPA) epidemiology and supporting the development of effective treatment strategies.

## 2 Materials and Methods

### 2.1 Sampling and Processing

Sputum and throat specimens were collected from children with CF between December 2023 and August 2024 at the Children's Medical Center, a referral hospital in Tehran, Iran. Samples were collected during episodes clinically consistent with CF pulmonary exacerbation/lung infection, defined as an acute worsening of respiratory symptoms (e.g., increased cough and/or sputum volume, dyspnea) and/or spirometric decline, accompanied by a clinician's decision to initiate antibiotic therapy and/or hospitalize the patient (Almulhem et al. 2024). Each sample was obtained from a distinct patient, with no repeated collections from the same individual. Both inpatients' and outpatients' specimens were examined for the isolation of *P. aeruginosa*. For precise identification of the bacterium, various conventional microbiological and biochemical tests were employed (Leber 2016). All samples identified as *P. aeruginosa* were then stored in tryptic soy broth (Merck) with 10% glycerol at −80°.

### 2.2 Antimicrobial Susceptibility Testing

Susceptibility of all isolates to antibiotics was determined using the Kirby–Bauer disc diffusion approach on Mueller–Hinton agar plates (Merck), consistent with the guidelines provided by the Clinical and Laboratory Standards Institute (CLSI) (CLSI 2024). The antibiotics tested (Mast Group Ltd., UK) included monobactams (aztreonam, 30 µg), cephems (ceftazidime, 30 µg; cefepime, 30 µg), fluoroquinolones (ciprofloxacin, 5 µg), carbapenems (imipenem, 10 µg; meropenem, 10 µg), and β‐lactam/β‐lactamase inhibitor combinations (piperacillin‐tazobactam, 100/10 µg). Quality control was performed with *P. aeruginosa* ATCC 27853. The criteria for identifying MDR involved resistance to at least one antibiotic from three or more distinct antimicrobial classes (Sahoo 2024), and CRPA was defined as resistance to at least one carbapenem (imipenem or meropenem). Nonsusceptibility was interpreted as intermediate or resistant according to CLSI guidelines.

### 2.3 Phenotypic Detection of Metallo‐β‐Lactamase (MBL)

The combined double‐disk synergy test was used to evaluate the production of MBL in CRPA. A suspension of each isolate was prepared to a 0.5 McFarland concentration and spread onto Mueller–Hinton agar plates. Two imipenem (10 µg) discs and two ceftazidime (30 µg) discs were placed 2 cm apart on the agar. For one of the discs corresponding to each β‐lactam antibiotic, 5 µL of a 0.5‐M ethylenediaminetetraacetic acid solution (pH 8.0) was applied. After incubating overnight at 37°C, a positive result for MBL production was indicated by an enhanced inhibition zone of 8 mm or greater around the combined disc compared with the individual antibiotic disc (Karampoor et al. 2022).

### 2.4 Detection of Carbapenemase‐Producing Genes

All isolates were screened for carbapenemase‐associated genes, including *blaIMP*, *blaKPC*, *blaNDM*, *blaOXA*, *blaSIM*, *blaSPM*, and *blaVIM*, using PCR assays. Primer sequences, annealing temperatures, and expected amplicon sizes for each target gene are provided in Table S1 (Han et al. 2022; Fan et al. 2023; Ma et al. 2024). The DNA templates were extracted using the phenol–chloroform method. The PCR amplification was executed in a 25‐µL reaction mixture, which included 12 µL of Taq DNA polymerase 2× Master Mix RED (amplicon), 9 µL of DNase/RNase‐free distilled water, 1 µL of 10 pM for both forward and reverse primers, and 2 µL of the DNA template. The PCR thermal cycling protocol incorporated an initial denaturation at 94°C for 10 min, followed by 40 cycles consisting of 30 s of denaturation at 94°C, annealing for 45 s at 50°C–55°C (depending on the specific primer), extension at 72°C for 50 s, and a concluding extension phase at 72°C for 7 min. PCR products were analyzed using agarose gel electrophoresis on a 1.5% agarose gel (Sigma) stained with DNA Gel Stain (Pishgam).

### 2.5 Molecular Typing by RAPD–PCR

All *P. aeruginosa* isolates from CF patients underwent RAPD–PCR typing using primer 272 (5ʹ‐AGCGGGCCAA‐3ʹ) at 0.4 mM in 25 µL reactions, as described earlier. Amplification included an initial denaturation (95°C, 5 min), 30 cycles of 94°C (1 min), 36°C annealing (1 min), and 72°C extension (5 min), with a final extension at 72°C (15 min) (Mamishi et al. 2023). To minimize technical variability, all reactions used a single master mix on one thermal cycler with standardized DNA templates (20–30 ng/µL) and *P. aeruginosa* ATCC 27853 as an internal control. Amplicons were separated on a 1.5% agarose gel (Sigma), and gel electrophoresis conditions (120 V, 60 min) were kept constant. The DNA band size was determined against a 100‐bp ladder (DM2300 ExcelBand, SMOBIO) and visualized with DNA Gel Stain. GelCompar II version 6.5 (Applied Maths) was used to analyze banding patterns, and clustering was executed with the UPGMA algorithm with a 30% distance cutoff (70% similarity), chosen based on literature precedent (Abdel‐Rhman and Rizk 2021), discriminatory power (Simpson's Diversity Index), and meaningful cluster sizes. This cutoff is an operational criterion used to summarize relatedness rather than a universal biological boundary. The distance matrix obtained from GelCompar II was used to generate a circular dendrogram in Python.

### 2.6 Data Analysis

Statistical analyses were conducted using IBM SPSS 25.0 and Microsoft Excel 365. Descriptive statistics included frequencies and percentages for categorical variables. Categorical associations were evaluated using Pearson's chi‐square or Fisher's Exact tests, with Cramer's *V* quantifying effect sizes (0.1, 0.3, and 0.5 indicating small, moderate, and large effects, respectively). Associations between carbapenemase gene coexistence and MDR and CRPA phenotypes were assessed using Spearman's rank correlation. Independent predictors of AMR were evaluated using multivariable binary logistic regression, adjusting for potential confounders; effect estimates were reported as adjusted odds ratios (AORs) and 95% confidence intervals (CIs). Statistical significance was set at two‐tailed *p* ≤ 0.05. The circular dendrogram was generated using Python 3.12 with NumPy, SciPy, Matplotlib, and Radial Tree libraries.

## 3 Results

### 3.1 Sample Characteristics

Microbiological and biochemical testing was performed on 672 clinical samples, including 209 sputum and 463 throat swabs, resulting in the isolation of 117 *P. aeruginosa* isolates. The overall prevalence of *P. aeruginosa* was 17.41%, with a breakdown of 21.5% in sputum samples and 15.5% in throat samples. Among these confirmed cases, 60 (51.3%) were female and 57 (48.7%) were male, with an average age of 12.1 ± 7.2 years, which were categorized into three age groups (Table 1). The distribution of *P. aeruginosa* isolates by hospital ward revealed some variations, with the outpatient department (OPD) lung clinic showing the highest prevalence at 63.2%, followed by the heart and lung ward at 15.4%, and the emergency ward at 14.6%.

**Table 1:** Distribution of CRPA and MDR by patient demographics.

Variable	Total isolates *n* (%)	Non‐CRPA *n* (%)	CRPA *n* (%)	Non‐MDR *n* (%)	MDR *n* (%)
*Gender*					
Male	57 (48.7)	47 (82.5)	10 (17.5)	45 (78.9)	12 (21.1)
Female	60 (51.3)	41 (68.3)	19 (31.7)	45 (75.0)	15 (25.0)
*Age*					
≤ 5 years	26 (22.2)	19 (73.1)	7 (26.9)	21 (80.8)	5 (19.2)
5–10 years	25 (21.4)	16 (64.0)	9 (36.0)	19 (76.0)	6 (24.0)
> 10 years	66 (56.4)	53 (80.3)	13 (19.7)	50 (75.8)	16 (24.2)
Total	117 (100)	88 (75.2)	29 (24.8)	90 (76.9)	27 (23.1)

Abbreviations: CRPA, carbapenem‐resistant *Pseudomonas aeruginosa*; MDR, multidrug resistant.

**Figure 1:**
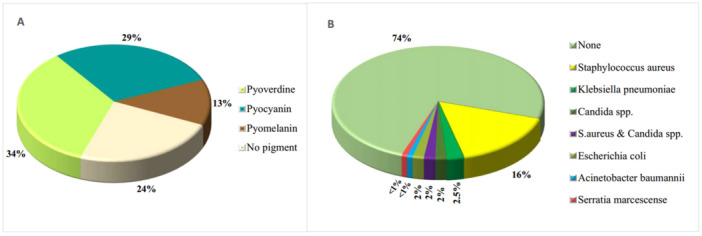
Pigment production and coinfection patterns among *Pseudomonas aeruginosa* infections. (A) Pigment distribution, with Pyoverdine and Pyocyanin as the most common and (B) coinfections were absent in most cases, but when present, *Staphylococcus aureus* predominated.

Different colony morphologies, including pigmentation and mucoid traits, were also identified among *P. aeruginosa* isolates. The analysis revealed that 55 isolates (47%) were mucoid and 62 isolates (53%) were nonmucoid. In the evaluation of pigment production, 40 samples exhibited pyoverdine pigment, 34 samples displayed pyocyanin pigment, and 15 samples presented pyomelanin pigment, while 28 samples did not produce any pigment (Figure 1A).

Pyoverdine production status was significantly associated with both MDR and CRPA phenotypes. Pyoverdine‐negative isolates (*n* = 77) exhibited higher resistance rates compared with pyoverdine‐positive isolates (*n* = 40): 29.9% versus 10.0% for MDR (*p* = 0.020; Cramer's *V* = 0.224), and 32.5% versus 10.0% for CRPA (*p* = 0.007; Cramer's *V* = 0.247). These inverse associations suggest that loss of pyoverdine production may be linked to acquisition or maintenance of AMR determinants. No significant associations were identified between pyocyanin or pyomelanin production and MDR or CRPA (all *p* > 0.05).

Among the 117 samples positive for *P. aeruginosa* isolates, coinfection with other bacteria was similarly assessed, revealing that 30 samples (25.6%) had coinfections, including 19 cases that were also positive for *Staphylococcus aureus*; notably, 14 of these cases were from throat samples and originated from OPD. The frequencies of other coinfections are presented in Figure 1B.

**Figure 2:**
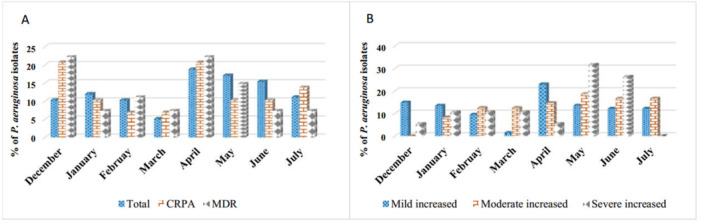
Seasonal trends in infection severity and resistance. (A) Resistance patterns revealed elevated CRPA and MDR rates in December and April. (B) Variations in infection severity show severe cases peaked in May and June, while mild increases were highest in April, highlighting critical seasonal spikes in both disease burden and antimicrobial resistance. CRPA, carbapenem‐resistant *Pseudomonas aeruginosa*; MDR, multidrug resistant.

The analysis of positive cases and the frequency of CRPA and MDR strains throughout the sampling period shows that April had the highest number of positive cases, followed by May and June (18.8%, 17.1%, and 15.4%). Additionally, in both December and April, the frequency of CRPA and MDR was noted at 20.7% and 22.2%, respectively (Figure 2A). Furthermore, significant variations in Colony Forming Unit were observed across the sampling periods, with both moderate and severe increases peaking in May and June (Figure 2B).

### 3.2 Antibiotic Resistance Profile

Antibiotic susceptibility testing for seven antimicrobial agents indicated that out of the 117 *P. aeruginosa* isolates, 6 (5.1%) were responsive to all antibiotics, and 94.9% showed nonsusceptibility to at least one. Resistance was notably high for aztreonam at 49.6%. In contrast, cefepime and imipenem exhibited lower resistance rates of 29.9% and 23.9%, respectively. Meanwhile, more than 75% of isolates were susceptible to ceftazidime, ciprofloxacin, meropenem, and piperacillin‐tazobactam (Table 2).

**Table 2:** Antimicrobial susceptibility patterns of *Pseudomonas aeruginosa* isolates in a CF patient.

	Total (*n* = 117)			Non‐CRPA (*n* = 88)			CRPA (*n* = 29)			*p* value
Antibiotic	R	I	S	R	I	S	R	I	S	
Aztreonam	58 (49.6)	37 (31.6)	22 (18.8)	33 (37.5)	34 (38.6)	21 (23.9)	25 (86.2)	3 (10.3)	1 (3.4)	0.013
Ceftazidime	15 (12.8)	1 (0.9)	101 (86.3)	1 (1.1)	—	87 (98.9)	14 (48.3)	1 (3.4)	14 (48.3)	< 0.001
Cefepime	35 (29.9)	16 (13.7)	66 (56.4)	13 (14.8)	14 (15.9)	61 (69.3)	22 (75.9)	2 (6.9)	5 (17.2)	< 0.001
Ciprofloxacin	16 (13.7)	12 (10.2)	89 (76.1)	6 (6.8)	8 (9.1)	74 (84.1)	10 (34.5)	4 (13.8)	15 (51.7)	< 0.001
Imipenem	28 (23.9)	3 (2.6)	86 (73.5)	—	3 (3.4)	85 (96.6)	28 (96.6)	—	1 (3.4)	< 0.001
Meropenem	8 (6.8)	5 (4.3)	104 (88.9)	—	2 (2.3)	86 (97.7)	8 (27.6)	3 (10.3)	18 (62.1)	< 0.001
Piperacillin‐tazobactam	22 (18.8)	6 (5.1)	89 (76.1)	6 (6.8)	3 (3.4)	79 (89.8)	16 (55.2)	3 (10.3)	10 (34.5)	< 0.001

*Note:* Variables are represented by No. (%). *p* Values represent comparisons between CRPA and non‐CRPA groups.

Abbreviations: CF, cystic fibrosis; CRPA, carbapenem‐resistant *Pseudomonas aeruginosa*; I, intermediate; R, resistant; S, sensitive.

Among the resistant isolates, 24.8% demonstrated CR status, while 23.1% exhibited MDR status. Additionally, 13.7% were identified as producers of MBL. Among MDR isolates, 11 (40.8%), 8 (29.6%), and 8 (29.6%) were associated with resistance across 3, 4, and 5 antibiotic classes, respectively. Resistance profiles ranged between 3 and 7 antibiotics, leading to the identification of 15 different patterns shown in Table 3.

**Table 3:** Antibiotic resistance profile in 27 MDR isolates.

Antibiotic profile	No. of antibiotic groups	No. (%)
ATM, CAZ, CPM, CIP, IMI, MEM, PTZ	5	4 (14.8)
ATM, CAZ, CPM, CIP, IMI, PTZ	5	1 (3.7)
ATM, CPM, CIP, IMI, PTZ	5	3 (11.1)
ATM, CAZ, CPM, IMI, MEM, PTZ	4	1 (3.7)
CAZ, CPM, CIP, IMI, MEM, PTZ	4	1 (3.7)
ATM, CAZ, CPM, IMI, PTZ	4	3 (11.1)
ATM, CAZ, CIP, IMI	4	1 (3.7)
ATM, CAZ, IMI, PTZ	4	1 (3.7)
ATM, CPM, IMI, PTZ	4	1 (3.7)
ATM, CPM, CIP	3	2 (7.4)
ATM, CPM, IMI	3	5 (18.5)
ATM, CPM, MEM	3	1 (3.7)
ATM, CPM, PTZ	3	1 (3.7)
ATM, CIP, PTZ	3	1 (3.7)
ATM, IMI, PTZ	3	1 (3.7)

Abbreviations: ATM, aztreonam; CAZ, ceftazidime; CPM, cefepime; CIP, ciprofloxacin; IMI, imipenem; MDR, multidrug resistant; MEM, meropenem; PTZ, piperacillin‐tazobactam.

**Figure 3:**
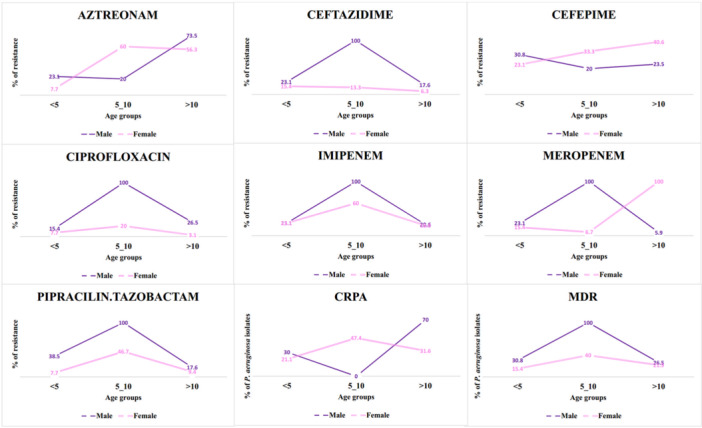
Resistance profiles of antibiotics by age and gender. Age‐ and sex‐stratified antibiotic resistance proportions and MDR/CRPA phenotypes. Aztreonam resistance increased significantly with age (*p* < 0.001, Bonferroni‐corrected). Meropenem showed marginal variation (*p* = 0.018, nonsignificant after correction). Other antibiotics and gender comparisons showed no significant differences. CRPA, carbapenem‐resistant *Pseudomonas aeruginosa*; MDR, multidrug resistant.

Differences in resistance levels were analyzed between genders within different age groups (Figure 3). Aztreonam resistance increased significantly with age (*χ* ^2^ = 18.87, *p* < 0.001, Bonferroni‐corrected), from 15.4% in children < 5 years to 65.2% in those > 10 years. Meropenem showed limited age‐related variation (*χ* ^2^ = 8.09, *p* = 0.018), which was not significant after correction. No significant age‐associated differences were observed for other antibiotics or MDR/CRPA phenotypes (all *p* > 0.05). No gender‐based differences were detected within age groups after Bonferroni adjustment (*α* = 0.00185; all *p* > 0.05).

Multivariable logistic regression models were constructed to identify predictors of AMR, adjusting for age group, gender, sample type, and hospital ward. Three models achieved statistical significance: aztreonam resistance (*χ* ^2^ = 24.84, *p* = 0.001), imipenem resistance (*χ* ^2^ = 14.19, *p* = 0.048), and CRPA (*χ* ^2^ = 14.20, *p* = 0.048). Age was the only significant predictor of aztreonam resistance, with children < 5 years demonstrating 87% reduced odds compared with those > 10 years (AOR = 0.130, CI 0.038–0.445). Hospital ward was the primary predictor for both imipenem resistance (AOR = 9.334, CI 1.764–49.397) and CRPA (AOR = 8.866, CI 1.678–46.862), with specific wards demonstrating substantially elevated odds of resistance. Gender and sample type showed no significant associations with resistance for any antibiotic tested (all *p* > 0.05).

### 3.3 CR Genes

**Figure 4:**
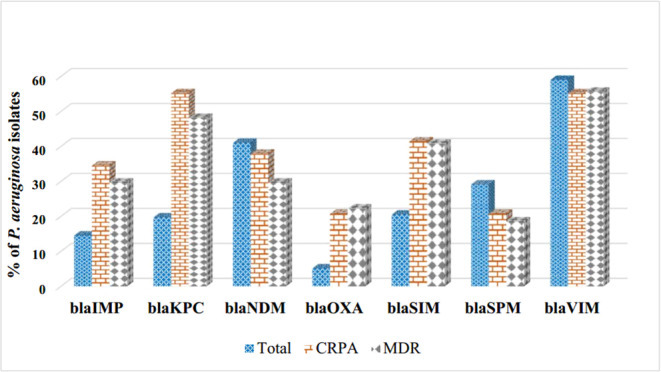
Distribution of carbapenemase genes and resistance patterns. Carbapenemase gene prevalence and associated resistance showed high rates of *blaKPC, blaVIM*, and *blaNDM* in carbapenem‐resistant *Pseudomonas aeruginosa* (CRPA) and multidrug‐resistant (MDR) *Pseudomonas aeruginosa* isolates. These findings emphasize the clinical impact of these genes in MDR.

Seven carbapenemase‐encoding genes were examined in 117 *P. aeruginosa* isolates via PCR. The analysis indicated that 102 (87.2%) of the isolates possessed at least one of the seven genes. The most frequently encountered gene was *blaVIM* (59%), followed by *blaNDM* (41%). Among carbapenemase genes detected in resistant isolates, *blaVIM* and *blaKPC* were the most prevalent. In CRPA isolates, both genes were detected at equal frequency (55.2% each), while in MDR isolates, *blaVIM* showed a slightly higher prevalence (55.6%) than *blaKPC* (48.1%). Other carbapenemase genes were detected at lower frequencies (Figure 4). Spearman's rank correlation analysis revealed significant positive associations between carbapenemase gene coexistence and both resistance phenotypes: MDR (*ρ* = 0.227, *p* = 0.014) and CRPA (*ρ* = 0.314, *p* = 0.001), with CRPA demonstrating a stronger correlation.

### 3.4 Molecular Typing

**Figure 5:**
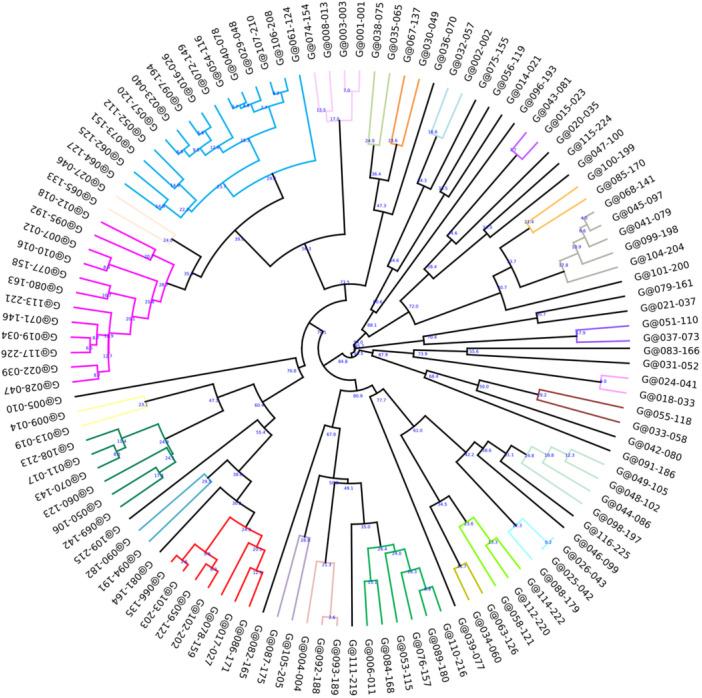
Dendrogram with color‐coded clusters. The dendrogram, derived from RAPD–PCR analysis of *Pseudomonas aeruginosa* strains isolated from CF children, illustrates cluster definition at a ≤ 30% dissimilarity threshold, with clusters distinguished by color. CF, cystic fibrosis; PCR, polymerase chain reaction; RAPD, random amplified polymorphic DNA.

RAPD–PCR analysis using primer 272 generated 1–9 amplicon bands ranging from 100 to 3000 bp across all isolates. Dendrogram analysis of *P. aeruginosa* isolates (Figure 5), applying a 30% distance cut‐off (≥ 70% similarity), identified 24 distinct genetic clusters, each comprising 2–15 members. The distribution included 12 clusters with 2 members, 4 clusters with 3 members, 2 clusters each containing 4 and 6 members, and individual clusters comprising 5, 7, 12, and 15 members. Overall, 95 isolates (81.2%) were grouped into clusters, while 22 isolates (18.8%) remained as unique genotypes (singletons), reflecting high genetic diversity (Simpson's Diversity Index = 0.9605). Notably, 11 of these clusters exhibited members with over 90% similarity, indicating highly related clonal isolates. Among these, samples G@025‐042 and G@026‐043, isolated from twin sisters, demonstrated nearly complete similarity (> 99%).

Epidemiological analysis revealed that the two dominant clusters exhibited temporal persistence throughout the study period, with isolates recovered from both throat and sputum samples across different clinical settings. This pattern suggests either sustained nosocomial transmission or a common environmental reservoir. Correlation analysis between cluster membership and clinical variables demonstrated that major clusters were not significantly associated with resistance phenotypes (MDR/CRPA) or sample types (throat vs. sputum). However, cluster assignment showed a statistically significant association with ward distribution (*χ* ^2^ = 74.95, df = 21, *p* < 0.001, CI 0.000–0.025), with a moderate effect size (Cramer's *V* = 0.462).

## 4 Discussion

The current study assesses the prevalence, AMR patterns, CR genes, and genetic diversity of *P. aeruginosa* isolates from pediatric CF patients at a major Iranian referral hospital, where referral of more severe or treatment‐experienced cases may contribute to higher observed MDR and CRPA rates compared with community‐based or broader multicenter cohorts.

Our findings revealed a 17.41% prevalence of *P. aeruginosa* among 51.3% females and 48.7% males with CF, which is consistent with other findings from Iran (Mamishi et al. 2023; Erfanimanesh et al. 2022). We also examined the phenotypic characteristics of *P. aeruginosa* isolates, noting differences in colony morphology and pigment production. Forty‐seven percent of isolates were mucoid, a trait commonly associated with virulence in respiratory infections. Mucoid strains of *P. aeruginosa* are known to produce alginate, which enhances their ability to persist in the lungs (Elfadadny et al. 2024; Erfanimanesh et al. 2022). Pyoverdine (34.2%) and pyocyanin (29%) production reflected their established roles in virulence and immune modulation. Notably, pyoverdine production showed significant inverse associations with both MDR and CRPA phenotypes, potentially reflecting metabolic trade‐offs where resistant isolates prioritize resistance determinants over energy‐intensive siderophore biosynthesis under antibiotic pressure (Elfadadny et al. 2024; Kang et al. 2019). This phenomenon warrants investigation as a potential rapid phenotypic screening marker for AMR.

Our study detected 25.6% of *P. aeruginosa* cases associated with other bacterial infections, particularly *S. aureus*. This finding underscores the complex nature of CF pulmonary infections, where polymicrobial infections can complicate diagnosis and treatment (King et al. 2022). Additionally, the seasonal trends observed in our study, with peak cases in April, May, and June, align with reports from other regions where seasonal variations in bacterial prevalence have been observed, possibly due to environmental factors (van Veen et al. 2025). Moreover, in both December and April, the frequency of CRPA and MDR increased. Such temporal trends may reflect antibiotic usage patterns, with increased use of broad‐spectrum antibiotics during certain seasons potentially driving resistance development (He et al. 2023).

Widespread antibiotic use, both with and without medical supervision, has led to the rise of MDR, creating significant therapeutic challenges. Our findings revealed that nearly 95% of *P. aeruginosa* isolates were nonsusceptible to at least one antibiotic, with notable resistance against aztreonam and cefepime. This is consistent with other studies reporting high resistance to β‐lactam agents, particularly in hospital‐acquired *P. aeruginosa* infections in pediatric populations (Gutiérrez‐Santana et al. 2022; Hafiz et al. 2023; Patil et al. 2023). Interestingly, higher susceptibility rates were noted for ceftazidime, ciprofloxacin, meropenem, and piperacillin‐tazobactam, which aligns with previous reports from the same center (Mamishi et al. 2023) and suggests that these antibiotics still offer efficacy against *P. aeruginosa* infections in pediatric patients.

The growing occurrence of CRPA strains represents a critical healthcare challenge, highlighting the urgent need for timely identification to ensure effective treatment and reduce nosocomial spread (WHO 2024). In the current study, the rates of CR (24.8%) and MDR (23.1%) are particularly concerning, as they are higher than previous reports (Mamishi et al. 2023; Mahmoudi et al. 2025). This increase may reflect intensified antibiotic selection pressure, enhanced surveillance sensitivity, or successful clonal expansion of resistant strains within the hospital environment, consistent with rising CR trends observed nationally (Rastegar‐Kashkouli et al. 2024), which highlights the urgent need for alternative treatment options and the implementation of strict antimicrobial stewardship protocols.

Age‐related resistance differences were limited after Bonferroni correction. Only aztreonam resistance increased significantly with age (*χ* ^2^ = 18.87, *p* < 0.001). Meropenem showed no significant age‐related variation after correction (*p* = 0.018), and no gender‐based differences were detected within any age group. These findings suggest that pediatric age may influence antibiotic susceptibility, potentially due to differences in pharmacokinetics, immune response, or the microbiome composition in different age groups (Kitano et al. 2025).

Molecular analysis revealed that 87.2% of isolates carried at least one carbapenemase gene, with *blaVIM* being the most prevalent, consistent with studies highlighting *blaVIM* in pediatric *P. aeruginosa* infections (Patil et al. 2023; Althaferi et al. 2025; Ngoi et al. 2021). The coexistence of *blaVIM* and *blaKPC* in both CRPA and MDR isolates suggests a complex, multifactorial genetic resistance with potential for horizontal gene transfer within hospital settings (Mohamed et al. 2023; Zheng et al. 2025).

However, phenotypic CR was observed in fewer isolates than genotypic carriage, which is biologically plausible given the multifactorial nature of CR in *P. aeruginosa* and the fact that gene presence does not ensure sufficient expression for phenotypic resistance (Diorio‐Toth et al. 2022). Spearman's analysis showed significant positive associations between carbapenemase gene coexistence and the CRPA phenotype, suggesting that multiple genes increase resistance likelihood. Conversely, some CR isolates lacked detectable carbapenemase genes, likely reflecting alternative mechanisms, such as porin loss, efflux overexpression, or AmpC derepression (Yang et al. 2025). Thus, gene detection reflects resistance potential rather than definitive CR.

Molecular typing revealed a diverse genetic population with 24 distinct clusters, of which 11 showed greater than 90% genetic similarity, indicating localized clonal spread (Van Belkum et al. 2007). However, significant ward‐cluster associations reflected ward‐specific distributions rather than hospital‐wide transmission. A substantial proportion of singleton isolates (18.8%) confirmed that most infections arose from independent sources, consistent with *P. aeruginosa*'s opportunistic nature and supporting diverse acquisition pathways rather than single‐source outbreaks.

Despite the environment being the primary source of CF pathogens, strong evidence supports patient‐to‐patient transmission occurring inside and outside hospitals, where hospitalization itself is associated with an elevated risk of acquiring mucoid *P. aeruginosa* (Mamishi et al. 2023; Reyle et al. 2025). This emphasizes the need for enhanced infection control measures to prevent outbreaks, especially in pediatric populations who may be more susceptible to hospital‐acquired infections.

This study has several limitations that warrant consideration. First, the single‐center design limits the generalizability of our findings to other pediatric CF populations, particularly across different geographic regions with varying antimicrobial prescribing patterns and infection control practices. Second, while RAPD–PCR provided a cost‐effective approach for molecular typing in our resource‐limited setting, this method has inherent reproducibility limitations compared with more discriminatory techniques, such as multilocus sequence typing (MLST) or whole‐genome sequencing (WGS). Third, the cross‐sectional design precludes assessment of temporal changes in resistance patterns or longitudinal colonization dynamics within individual patients. Fourth, we did not evaluate clinical outcomes or treatment responses associated with specific resistance phenotypes or genotypes, which limits our ability to assess the clinical impact of MDR and CRPA infections. Finally, the relatively small sample size may have limited statistical power to detect weaker associations between certain risk factors and resistance outcomes. Future multicenter, longitudinal studies employing MLST/WGS would provide more comprehensive insights into the molecular epidemiology and transmission dynamics of *P. aeruginosa* in the pediatric CF population.

## 5 Conclusion

Our study reveals a substantial burden of antimicrobial‐resistant *P. aeruginosa* among pediatric CF patients at a major Iranian referral hospital, with 24.8% CR and 23.1% MDR. The widespread distribution of carbapenemase genes (87.2% of isolates), particularly *blaVIM* and *blaKPC*, combined with evidence of clonal transmission across hospital wards, underscores urgent challenges in this setting. Age‐dependent resistance patterns and significant ward‐cluster associations provide actionable targets for antimicrobial stewardship interventions at this tertiary care center. These findings emphasize the need for comprehensive multicenter surveillance studies, enhanced infection control measures, and investigation of alternative therapeutic strategies, including bacteriophage therapy and engineered antimicrobials, to address resistance in the pediatric CF population.

## Author Contributions

**Erfaneh Jafari:** writing – original draft, conceptualization, data curation, formal analysis, investigation, methodology, visualization. **Babak Pourakbari:** writing – review and editing, supervision, validation. **Mohammad Reza Asadi Karam:** writing – review and editing, investigation, data curation. **Reza Azizian** and **Mohammad Reza Modaresi:** writing – review and editing, investigation. **Setareh Mamishi:** writing – review and editing, supervision, funding acquisition. All authors read and approved the final manuscript.

## Ethics Statement

Approval for the sampling protocols was granted by the Ethics Committee of Tehran University of Medical Sciences (IR.TUMS.CHMC.REC.1402.136), and parental or guardian informed consent was acquired prior to the data collection.

## Consent

The authors have nothing to report.

## Conflicts of Interest

None declared.

## Supporting information


**Table S1:** Primer Sequences and PCR Products Size.

## Data Availability

The data that support the findings of this study are available from the corresponding author upon reasonable request.
