# Supplementary material for: The Burden of Antimicrobial‐Resistant Pseudomonas aeruginosa Isolates in Children With Cystic Fibrosis: Molecular Characterization and Genotyping Analysis
Source: Microbiologyopen. 2026 Jan 28;15(1):e70217. doi: 10.1002/mbo3.70217 (PMC12852059; doi:10.1002/mbo3.70217)
Supplement: Supplementary file 1 — Table S1: Primer Sequences and PCR Products Size. [file MBO3-15-e70217-s001.docx]

**Table S1 Primer Sequences and PCR Products Size**

| **Target Gene** | **Amplicon Size (bp)** | **Annealing Tm** | **Primer Sequence (5′ to 3′)** | **Reference** |
| --- | --- | --- | --- | --- |
| *blaIMP* | 587 | 50°C | F: GAAGGCGTTTATGTTCATAC | [Han et al. 2022] |
|  |  |  | R: GTACGTTTCAAGAGTGATGC |  |
| *blaKPC* | 888 | 55°C | F: ATGTCACTGTATCGCCGTCTA | [Fan et al. 2023] |
|  |  |  | R: TTACTGCCCGTTGACGCCCAA |  |
| *blaNDM* | 621 | 52°C | F: GGTTTGGCGATCTGGTTTTC | [Ma et al. 2024] |
|  |  |  | R: CGGAATGGCTCATCACGATC |  |
| *blaOXA* | 426 | 52°C | F: GCGTGGTTAAGGATGAACAC | [Ma et al. 2024] |
|  |  |  | R: CATCAAGTTCAACCCAACCG |  |
| *blaSIM* | 571 | 52°C | F: TACAAGGGATTCGGCATCG | [Han et al. 2022] |
|  |  |  | R: TAATGGCCTGTTCCCATGTG |  |
| *blaSPM* | 271 | 52°C | F: AAAATCTGGGTACGCAAACG | [Ma et al. 2024] |
|  |  |  | R: ACATTATCCGCTGGAACAGG |  |
| *blaVIM* | 390 | 52°C | F: GATGGTGTTTGGTCGCATA | [Ma et al. 2024] |
|  |  |  | R: CGAATGCGCAGCACCAG |  |
